# A novel mouse model recapitulating the MMR-defective SCLC subtype uncovers an actionable sensitivity to immune checkpoint blockade

**DOI:** 10.1007/s00432-024-05942-9

**Published:** 2024-11-14

**Authors:** Olta Ibruli, France Rose, Filippo Beleggia, Anna Schmitt, Maria Cartolano, Lucia Torres Fernandez, Julia Saggau, Debora Bonasera, Martha Kiljan, Gokcen Gozum, Luca Lichius, Jiali Cai, Li-na Niu, Manoela Iannicelli Caiaffa, Jan M. Herter, Henning Walczak, Gianmaria Liccardi, Holger Grüll, Reinhard Büttner, Graziella Bosco, Julie George, Roman K. Thomas, Kasia Bozek, Hans Christian Reinhardt, Grit S. Herter-Sprie

**Affiliations:** 1 Department of Translational Genomics, Faculty of Medicine, University Hospital Cologne, University of Cologne, Cologne, Germany; 2 Department I of Internal Medicine, Faculty of Medicine, University Hospital Cologne, University of Cologne, Cologne, Germany; 3 Center for Molecular Medicine Cologne, Faculty of Medicine, University Hospital Cologne, University of Cologne, Cologne, Germany; 4 Institute for Biomedical Informatics, Faculty of Medicine, University Hospital Cologne, University of Cologne, Cologne, Germany; 5 Mildred Scheel School of Oncology Aachen Bonn Cologne Düsseldorf (MSSO ABCD), Faculty of Medicine, University Hospital Cologne, University of Cologne, Cologne, Germany; 6 Institute of Virology, Faculty of Medicine, University Hospital Cologne, University of Cologne, Cologne, Germany; 7 Institute of Biochemistry I, Center for Biochemistry, Faculty of Medicine, University Hospital Cologne, University of Cologne, Cologne, Germany; 8 Cologne Excellence Cluster on Cellular Stress Responses in Aging-Associated Diseases, University of Cologne, Cologne, Germany; 9 Department II of Internal Medicine, Faculty of Medicine, University Hospital Cologne, University of Cologne, Cologne, Germany; 10 Department of Radiation Oncology and Cyberknife Center, Faculty of Medicine, University Hospital of Cologne, University of Cologne, Cologne, Germany; 11 Department of Radiation Oncology, MVZ Prof. Dr. Uhlenbrock und Partner, Gesundheitscampus Josephs-Hospital, Warendorf, Germany; 12 Center for Cell Death, Cancer, and Inflammation (CCCI), UCL Cancer Institute, University College London, London, UK; 13 Institute for Diagnostic and Interventional Radiology, Faculty of Medicine, University Hospital Cologne, University of Cologne, Cologne, Germany; 14 Institute of Pathology, Faculty of Medicine, University Hospital Cologne, University of Cologne, Cologne, Germany; 15 Department of Otorhinolaryngology, Faculty of Medicine, University Hospital of Cologne, University of Cologne, Cologne, Germany; 16 Department of Hematology and Stem Cell Transplantation, University Hospital Essen, Essen, Germany; 17 West German Cancer Center, University Hospital Essen, Essen, Germany; 18 DKTK Partner Site Essen/Düsseldorf, University Hospital Essen, Essen, Germany; 19 Center for Molecular Biotechnology, University Hospital Essen, Essen, Germany; 20 Department of Medical Oncology, Fachklinik Hornheide, Münster, Germany

**Keywords:** Small cell lung cancer, Immune checkpoint inhibitor, Tumor mutational burden, Mismatch repair deficiency, Genetically engineered mouse model

## Abstract

**Purpose:**

Small cell lung cancer (SCLC) has an extremely poor prognosis. Despite high initial response rates to chemotherapy and modest survival improvements with the addition of immune checkpoint inhibitors (ICI), almost all patients experience relapse and fatal outcomes. Recent genomic insights uncovered extensive molecular heterogeneity in addition to the almost uniform loss of *RB1* and *TRP53*. Additionally, defective DNA mismatch repair (MMR) has recently been described in some SCLC cases. Here, we generated a novel SCLC mouse model capturing MMR deficiency and assessed immunotherapy responses.

**Methods:**

We developed an MMR-deficient genetically engineered mouse model (GEMM) of SCLC by introducing a conditional *Msh2* gene, crucial for maintaining MMR integrity, into the standard *Rb1*^*fl/fl*^;*Trp53*^*fl/fl*^ (RP) model. Genomic characteristics and preclinical therapy responses were evaluated by focusing on overall survival and whole exome sequencing (WES) analyses.

**Results:**

MMR-defective SCLC tumors (*Rb1*^*fl/fl*^;*Trp53*^*fl/fl*^;*Msh2*^*fl/fl*^ (RPM)) developed later than tumors in MMR-proficient mice. However, the time from tumor manifestation to death of the affected animals was substantially shortened (median survival 55 days in RP vs. 46.5 days in RPM), indicating increased aggressiveness of MMR-defective tumors. RPM tumors exhibited MMR deficiency, high tumor mutational burden (TMB), and an elevated load of candidate neoantigens, compared to RP lesions (*p* = 0.0106), suggesting increased immunogenicity. Importantly, the overall survival of RPM animals was significantly improved when exposed to ICI.

**Conclusion:**

We propose a novel RPM mouse model as a suitable system to mimic MMR-defective SCLC and tumors with high TMB. We provide *in vivo* evidence that *Msh2* deficiency enhances ICI sensitivity. These findings could contribute to stratifying SCLC patients to immunotherapy, thereby improving treatment outcomes.

**Supplementary Information:**

The online version contains supplementary material available at 10.1007/s00432-024-05942-9.

## Introduction

Small cell lung cancer (SCLC) comprises 15% of lung tumors and is characterized by rapid proliferation and early dissemination of metastases. While patients with SCLC are almost uniformly responsive to initial chemotherapy, the 5 year survival rate remains < 10% since most patients will inevitably relapse (Nicholson et al. 2016). Despite recent advancements in improving therapeutic strategies, such as the addition of immune checkpoint inhibitors (ICI), atezolizumab or durvalumab, to chemotherapy as the firstline treatment, only a modest increase in overall survival by approximately two months was achieved (Horn et al. 2018; Dingemans et al. 2021; Paz-Ares Lancet 2019). Additionally, the clinical response varies substantially among patients, and it is still unclear which patients may benefit from the addition of ICI.

Comprehensive studies on deciphering the genomic complexity of SCLC have identified highly recurrent alterations in the *RB1* and *TRP53* tumor suppressor genes, which occur in almost all SCLC cases (George et al. 2015, 2024; Sivakumar et al. 2023). Although SCLC is typically associated with massive tobacco smoke exposure (Wang et al. 2023), emerging evidence strongly suggests that approx. 15% of clinical cases harbor a predominant DNA mismatch repair (MMR) mutational signature (Liu et al. 2024). MMR defects result in genomic instability and a high tumor mutational burden (TMB) (Dietlein et al. 2014a). MMR has been shown to influence not only tumorigenesis, but also the response to immunotherapy by increasing the sensitivity of solid tumors to immune checkpoint inhibition (Le et al. 2017). In fact, the FDA has approved ICI therapy for cancers with MMR deficiency, underscoring the clinical significance of this biomarker (fda & cder, n.d.; Marcus et al. 2019). Similarly, recent efficacy data observed in patients with SCLC revealed that a high TMB provided greater clinical benefit when treated with a combination of two ICI agents (Hellmann et al. 2018). At this point, further validation of the impact of the mutational burden is needed to understand mechanisms mediating ICI efficacy.

Identifying mutationally-defined SCLC subtypes that respond distinctly to immunotherapy is, at this point, challenging since most patients present with an extensive stage disease that complicates in-depth studies on tumors. Furthermore, all patients are currently treated with a combination of chemotherapy and ICI, which complicates dissecting the effect of ICI (Rudin et al. 2021). Although genetically modified mice have provided important insights into the molecular features of the malignancy, current SCLC preclinical models do not fully recapitulate the human disease (Oser et al. 2024). The most commonly used mouse models mirror the conditional loss of *Rb1* and *Trp53* (referred to as the RP model, for *Rb1*^*fl/fl*^;*Trp53*^*fl/fl*^) and key histological and genome alterations found in patients (Meuwissen et al. 2003; McFadden et al. 2014), but fail to capture the high TMB that is typically observed in human SCLC.

Here, we introduce a novel SCLC mouse model harboring an MMR deficiency mutational signature and explore the impact of this genetic makeup on tumor aggressiveness and response to checkpoint blockade. We adapted the RP mouse model by breeding in a conditional *Msh2* knockout gene (*Rb1*^*fl/fl*^;*Trp53*^*fl/fl*^;*Msh2*^*fl/fl*^ (RPM)) using a previously published *Msh2* allele (Kucherlapati et al. 2010). *Msh2* is crucial to the MMR pathway by recognizing and binding to mismatched nucleotides during DNA replication, recruiting other MMR proteins to excise the error, and thus maintaining genomic stability. Upon deletion of *Msh2*, microsatellite instability (MSI) is induced (De Wind et al. 1995; Dietlein et al. 2014a). MSI induction leads to increased genomic instability and has significant implications for cancer treatment. Specifically, an MSI-high status in colorectal cancer was strongly associated with favorable survival outcomes in patients treated with pembrolizumab (a PD-1 blocking antibody) (Le et al. 2015). This finding prompted the FDA to approve MSI/MMR as a predictive biomarker for the pembrolizumab treatment of adult and pediatric patients with metastatic or unresectable solid tumors (Marcus et al. 2019; Wang et al. 2021). Through genomic and survival analyses, we investigated the impact of *Msh2* deletion on SCLC tumor development, mutational patterns, and ICI response. We detected higher tumor aggressiveness, increased TMB, a predominant MMR mutational signature, and more candidate neoantigens in RPM tumors compared to tumors derived from the RP model. Importantly, in vivo treatment uncovered an increased ICI sensitivity in RPM tumor-bearing mice compared to the RP parental strain. Our findings offer insights into potential patient stratification strategies and might ultimately help clinicians design informed patient-tailored clinical studies.

## Materials and methods

### Experimental mice

Animal experiments in this study were approved by the local Ethics Committee of Animal Experiments authorities (LANUV, North Rhine-Westphalia, Germany) under license number 81-02.04.2019-A491. All mice were maintained according to FELASA recommendations and in compliance with the European Union and German guidelines. Mice were bred and housed up to five per cage in individually ventilated cages (IVC), with a 12-hour light/dark cycle and a temperature of 20–22 °C. This study utilized the standard SCLC mouse line harboring *Rb1*^*fl/fl*^, where exons 18 and 19 are flanked by *loxP* sites, and *Trp53*^*fl/fl*^, where *loxP* sites enclose exons 2–10. This model is well-established for recapitulating SCLC and is commonly referred to as the RP model (Meuwissen et al. 2003). To induce MMR deficiency in the RP background, we introduced a conditional *Msh2* gene, generating the RPM model. This was achieved by mating the RP mice with *Msh2*^*LoxP/LoxP*^ mice, an *Msh2*-deficient intestinal cancer mouse carrying an *Msh2* genomic fragment with a flanked exon 12 (Kucherlapati et al. 2010), and then intercrossing to obtain the *Rb1*^*fl/fl*^;*Trp53*^*fl/fl*^;*Msh2*^*fl/fl*^ genetic background.

### Processing of whole exome sequencing (WES) data

Sequencing reads were aligned to the ensembl mouse reference GRCm39 using BWA v.0.7.17 (Li and Durbin 2009). PICARD v2.26 and samtools v 1.13 were used to mark and exclude PCR duplicates. Mutect2 from the Genome Analysis ToolKit (GATK) v4.2.1.0 was used to call somatic mutations using a panel of normals generated with 14 healthy control samples (Depristo et al. 2011). The resulting variants were filtered using GATK FilterMutectCalls, but without the filter for slippage. Variants identified in more than one sample or in dbsnp were excluded. Loci covered at a depth of less than 30 were also excluded. The TMB was calculated as the number of mutations/million bases covered at a depth of 30 or more.

### Estimation of the Msh2 recombination efficiency

The read counts for each exon were calculated using GATK CollectReadCounts over the exons included in the GENCDE vM27 reference. The counts of the floxed *Msh2* exon 12 were first normalized to the counts of the other *Msh2* exons and this ratio was then normalized to that of control mice of the RP genotype to derive the estimated recombination efficiency as the copy number of exon 12. For visualization (Extended Data Fig. 1a–f), the coverage of individual bases within the *Msh2* gene was extracted using GATK pileup v4.2.10. The predicted coverage profile of exon 12 in the absence of recombination (copy number = 2) was calculated using control mice of the RP genotype by dividing the coverage of individual exon 12 bases to the median coverage of bases within other exons. For each sample, this ratio was then multiplied by the median coverage of bases within non-floxed exons in the same sample.

### Mutational signature analysis

WES data was used to extract the genomic context of the single nucleotide variants (SNV) (i.e., the triplets) utilizing a customized Python script and the Mus_musculus.GRCm39 assembly as the reference genome. Mutational signature analysis was performed using SigProfilerAssignment (v0.0.24) and the mouse signature COSMIC_v3 as a reference set panel (Alexandrov et al. 2020). All resulting signatures with an activity > 0 across all samples were selected. To assess the activity of MMR deficiency signatures, SBS15 and SBS21 mutational signatures were considered. Statistical analyses were conducted using the Kruskal-Wallis statistical test.

### HLA-I binding prediction

The WES output was used to create a library of mutant coding sequences using an in-house pipeline, including missense, in-frame, and frameshift mutations. For missense and in-frame mutations, mutant sequences were trimmed to contain the mutation in a centered position flanked by 3’ and 5’ short sequences of 13 basepairs. Frameshift mutant sequences were trimmed only in the 3’ direction while preserving the whole frameshift in the 5’ end. This trimming strategy allows for the prediction of HLA-I binders of all possible sizes fitting the HLA-I peptide pocket (8–14 mers). Trimmed peptide sequences were used as input into the immune epitope database (IEDB) resource NetMHCpan (ver. 4.1) tool for HLA-I binding prediction (Reynisson et al. 2021). For simplicity, HLA-I binders were predicted only for the murine H2kb allele and 9 bp peptide length (9 mers), the most common binder size. Binders were selected based on a percentage rank below 2 and categorized as strong binders (rank < 0.5) and weak binders (0.5 < rank < 2). Statistical analyses were performed using the Kruskal-Wallis statistical test.

### Treatment cohorts

After MRI confirmation of tumor growth and sufficient tumor burden within the 5–20 mm^3^ range, mice were randomly assigned to different therapy regimens. Compound solutions were administered as follows: The anti-PD-1 antibody RMP1-14 (BioXCell) was administered intraperitoneally (i.p.) at 200 mg/mouse twice a week for three weeks or three times per week until termination criteria were met. Etoposide (Hexal) was injected i.p. at 8 mg/kg on days 1, 2, and 3 of a 14-day cycle, while Cisplatin (Accord) was given i.p. at 4 mg/kg on day 1 of a 14-day cycle. Phosphate-buffered saline (PBS) was used as the vehicle control for comparison. Survival analysis was recorded by classifying as events only the animals that succumbed to the disease or were euthanized due to predefined termination criteria. Animals terminated for unrelated reasons (e.g., non-malignancy weight loss or injuries caused by cage mates) were excluded from the analysis. The p-values were calculated using the Mann-Whitney statistical test.

## Results

### MMR deficiency is associated with enhanced SCLC aggressiveness in vivo

To date, translational studies in SCLC are mainly restricted to the RP mouse model, driven by conditional biallelic inactivation of the *Rb1* and *Trp53* tumor suppressor genes (Meuwissen et al. 2003), which are almost universally mutated across SCLC patients (George et al. 2015, 2024). However, existing preclinical tools do not fully reflect the genomic complexity of human disease, which includes high TMB, among other characteristics. Moreover, the current models only partially cover the emerging transcriptomic and genetic subtypes of SCLC. For instance, recent research efforts have suggested that SCLC comprises distinct molecular subtypes based on the expression of *ASCL1*,* NEUROD1*,* POUF2F3*, or *YAP1* transcription factors translating into potentially distinct therapeutic vulnerabilities (Rudin et al. 2019; Ireland et al. 2020). Additionally, a largescale gene expression analysis of human tumors classified patients into smoking-dominant, MMR-dominant, and APOBEC-dominant subtypes (Liu et al. 2024).

**Fig. 1:**
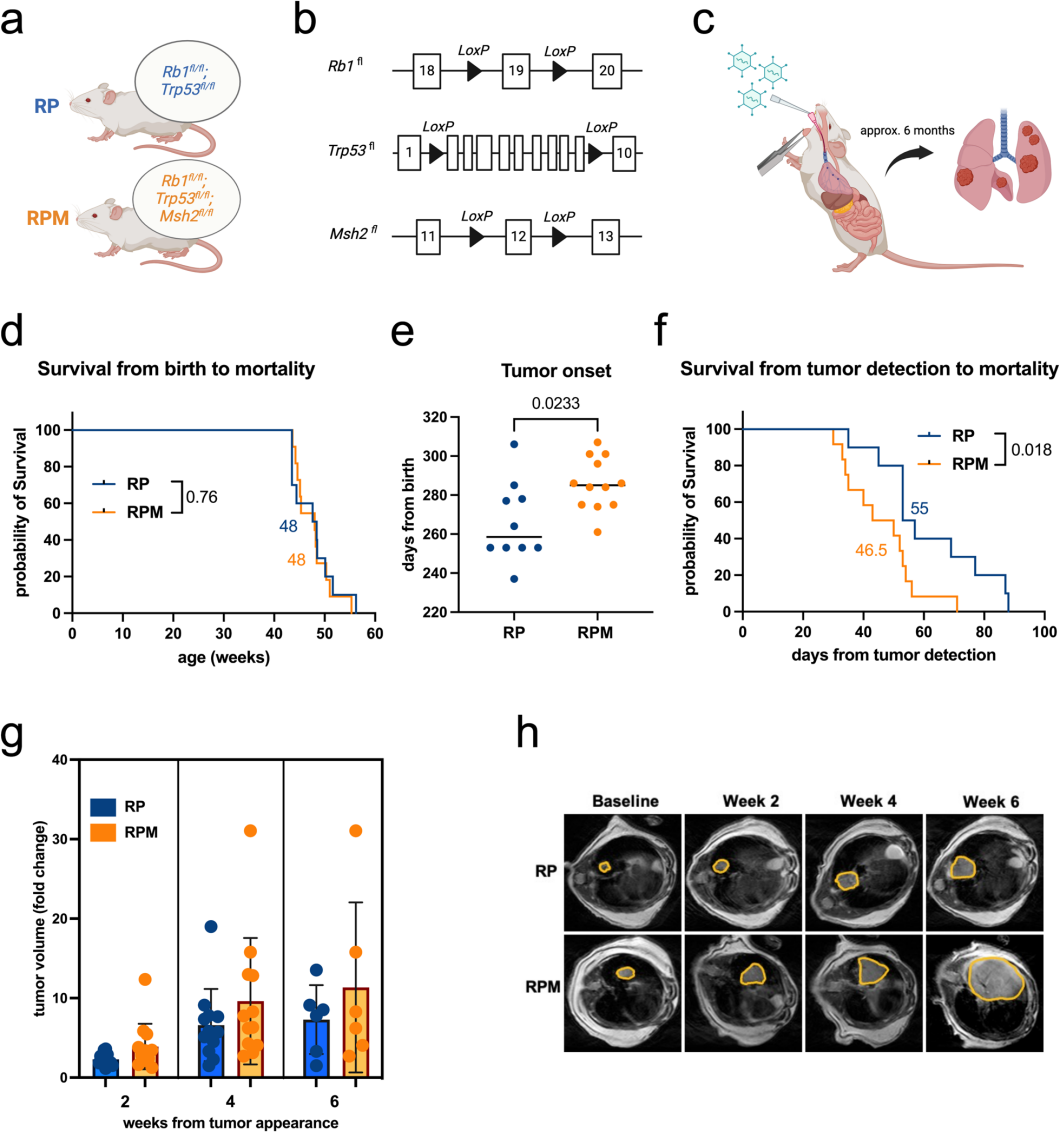
**RPM tumors reveal enhanced aggressiveness compared to RP lesions.** **a** Schematic of the RP and RPM mice with different genetic backgrounds. **b** Schematic of *Rb1*, *Trp53,* and *Msh2* alleles in the mouse models. **c** Induction of lung tumors via intratracheal inhalation of Adeno-Cre virus. **d** Survival curves from birth of RP (n  = 10, median 48 weeks) and RPM mice (n  = 11, median 48 weeks). **e** Tumor onset in RP (n  = 10, median 258.5 days) and RPM mice (n  = 12, median 285 days). **f** Overall survival determined from the timepoint of tumor onset of RP (n  = 10, median 55 weeks) and RPM mice (n  = 12, median 46.5 weeks). **g** Fold change in tumor volumes determined by quantifying segmented tumors in MRI scans at week 2 (n  = 12 RP, n  = 13 RPM), week 4 (n  = 12 RP, n  = 12 RPM), and week 6 (n  = 6 RP, n  = 6 RPM) after tumor detection. **h** Exemplary MRI scans of RP and RPM lung tumors as in (**g**). The figure was produced using licensed Biorender.com. Logrank (MantelCox) statistical test (**d**, **f**) and Mann-Whitney statistical t-test (**e**, **g**)

We, therefore, aimed to model a tumor with a high mutational burden. Since *Msh2* is a critical component of the MMR pathway, we introduced a conditional *Msh2* knockout allele into the RP background (Kucherlapati et al. 2010) (Fig. 1a, b). Lung tumors were induced by intratracheal instillation of Adeno-CMV-Cre, which led to the combined loss of all *Rb1*, *Trp53*, and *Msh2* alleles in the infected cells (Fig. 1c). Tumor manifestation was detected approximately six months following Adeno-CMV-Cre exposure and bi-weekly MRI scanning was utilized to track tumor development. We confirmed the loss of *Msh2* in tumors through coverage plots of WES data, which demonstrated successful Cre-mediated recombination of *Msh2* exon 12 in RPM mice, consistent with the expected coverage profiles for heterozygously and homozygously recombined alleles (Extended Data Fig. 1a–f). Histopathological examination of harvested lung lesions confirmed typical histological features of human SCLC in RPM tumors, such as dense tissue with small round to fusiform cells with scant cytoplasm and granular nuclear chromatin, which is also similar to tumors from RP mice (Raso et al. 2021; Meuwissen et al. 2003) (Extended Data Fig. 1g). To evaluate the impact of MMR deficiency on tumor development, we monitored the overall survival and tumor volume progression of RPM and RP animals. We observed no survival differences (Fig. 1d); however, tumor onset in RPM animals was significantly delayed compared to the RP parental strain with a median onset of 285 days in RPM animals vs. 258.5 days in RP animals (*p* = 0.0233) (Fig. 1e). Notably, RPM mice exhibited significantly decreased survival following initial tumor onset with a median survival of 46.5 days compared to 55 days in RP mice (*p* = 0.018) (Fig. 1f). MRI scans revealed no significant tumor volume differences, although RPM tumors tended to be larger than RP lesions at any given time (Fig. 1g, h and Extended Data Fig. 1h). Both models rarely developed metastases, which, when present, were confined to the liver (Extended Data Fig. 1i). Altogether, our findings indicate that incorporating an *Msh2* deletion into the RP background enhances the aggressiveness of SCLC tumors.

### *Msh2* deletion in the RP background results in high TMB and genomic instability

**Fig. 2:**
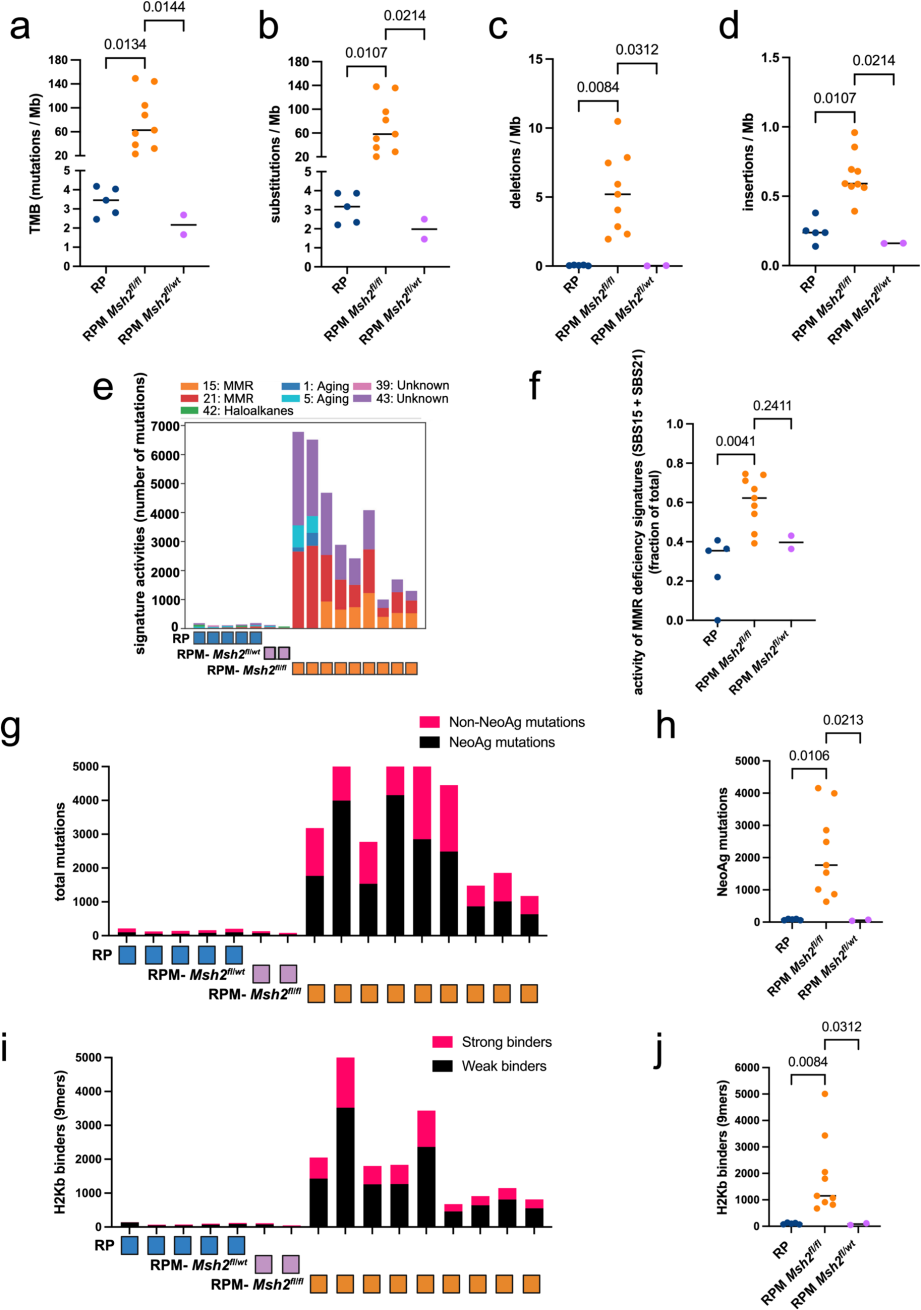
*Msh2* deletion on the RP background increases the TMB, exhibits an MMR mutation signature, and harbors significantly more neoantigens.  **a** Analysis of the TMB extent by WES. Identification of frameshift **b** substitutions, **c** deletions, and **d** insertions in the RP, RPM *Msh2*^fl/fl^ , and RPM *Msh2*^fl/wt^ background. **e** Assessment of mutational signatures by WES in RP and RPM animals based on COSMIC. **f** Statistical analysis of the activity of MMR deficiency signatures (SBS15 and SBS21) demonstrated as a fraction of the total in RP, RPM *Msh2*^fl/fl^ , and RPM *Msh2*^fl/wt^ lesions. **g** Assessment of neoantigen and non-neoantigen mutations, and **h** the corresponding statistical analysis. **i** Assessment of strong and weak H2Kb binders, and **j** the corresponding statistical analysis. All analyses are performed in n  = 5 RP, n  = 9 RPM with *Msh2*^fl/fl^, and n  = 2 RPM *Msh2*^fl/wt^. The figure was produced using licensed Biorender.com. Kruskal-Wallis statistical test

To assess the impact of *Msh2* loss on the genomic landscape of RPM tumors and to verify the presence of the desired engineered deletions, we conducted WES on harvested lung lesions. Among 11 RPM subjects derived from mating RP and *Msh2*^*LoxP/LoxP*^ mice (Meuwissen et al. 2003; Kucherlapati et al. 2010), 9 exhibited a homozygous loss of *Msh2* (*Msh2*^*fl/fl*^), while 2 had a mono-allelic loss (*Msh2*^*fl/wt*^) (Extended Data Fig. 2a). We determined the TMB by measuring the total number of mutations per million bases for each tumor. RPM tumors with *Msh2*^*fl/fl*^ showed an 18-fold greater TMB than the RP (*p* = 0.0134) and RPM tumors with heterozygous loss of *Msh2* (*p* = 0.0144), indicating that a complete loss of *Msh2* is necessary to increase the mutational load (Fig. 2a). Additionally, frameshift substitutions, deletions, and insertions were more common in RPM vs. RP tumors (Fig. 2b–d). To identify mutational MMR deficiency signatures, we performed mutational signature analysis based on the Catalogue of Somatic Mutations in Cancer (COSMIC). MMR deficiency signatures indicate the presence of deficiencies in DNA mismatch repair, typically represented by SBS15 and SBS21 mutational signatures, while MMR signature activities refers to the relative contribution of mutations associated with mismatch repair deficiency signatures among all mutations detected in a given tumor. We confirmed a predominant MMR deficiency signature in RPM mice in which *Msh2* was homozygously deleted. Of note, *Msh2* loss was not evident in tumors from RP mice (Extended Fig. 1a–b) and the MMR deficiency signature was substantially less dominant (*p* = 0.0041) (Fig. 2e, f). Yet, the detection of the MMR deficiency signature activities even in RP tumors, further indicates that MMR deficiency might be a selected event in SCLC. RPM tumors with heterozygous loss of *Msh2* displayed an MMR deficiency signature activity level comparable to RP tumors (Fig. 2e, f). Subsequently, to assess the immunogenic potential of mutations, we performed neoantigen prediction using the HLA-I binding tool from the immune epitope database (Reynisson et al. 2021). Analysis of mutant peptide sequences derived from WES revealed that RPM subjects had significantly more HLA-I binders than RP samples, with heterozygous RPM samples falling within the RP range. This was evidenced by a significantly higher number of neoantigenic mutations (*p =* 0.0106) and both strong and weak H2Kb binders (*p =* 0.0084) (Fig. 2g–j and Extended Data Fig. 2b). These results strongly suggest higher immunogenicity of RPM tumors, compared to RP lesions or subjects with a heterozygous loss of *Msh2*. Overall, WES analysis demonstrated that the homozygous loss of *Msh2* in the RP background significantly increased the TMB, MMR mutational signature, and candidate neoantigenic load, resembling the MMR-defective subset of human SCLC (Liu et al. 2024).

### RPM tumors display increased sensitivity to immune checkpoint inhibitors

**Fig. 3:**
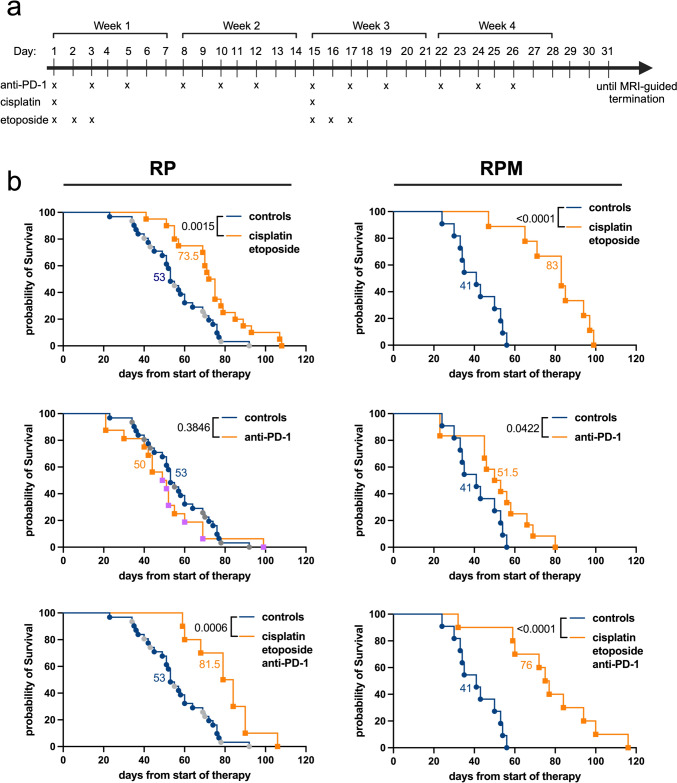
**RPM tumors display increased sensitivity toward ICI treatment.**  **a** Preclinical treatment schedule for RP and RPM tumor-bearing mice. **b** Kaplan-Meier survival curves for RP mice (left column) and RPM mice (right column) treated with: cisplatin/etoposide (*n*  = 20 RP and *n*  = 9 RPM), anti-PD-1 antibody (*n*  = 16 RP and *n*  = 12 RPM), a combination of cisplatin/etoposide and anti-PD-1 antibody (*n*  = 10 RP and *n*  = 10 RPM), and vehicle control (*n*  = 31 RP and *n*  = 11 RPM). Cisplatin was dosed at 4 mg/kg, i.p., and etoposide was dosed at 8 mg/kg, i.p. Two dosing schedules were applied for the anti-PD-1 antibody: three times/week until termination criteria (orange) and twice/week for a total of three weeks (purple). The figure was produced using licensed Biorender.com. Log-rank (Mantel-Cox) statistical test

To determine the effects of the *Msh2* deletion on ICI sensitivity, tumor-bearing animals were treated with an anti-PD-1 inhibitor upon MRI scanning-confirmed tumor manifestation. A pre-established preclinical dose was administered, with treatments continuing for three weeks or until predefined termination criteria were met. Additionally, treatments with cisplatin/etoposide alone and combined with ICI were included, representing the first-line treatment for patients with SCLC (Fig. 3a). As expected, since SCLC is initially highly responsive to chemotherapy, chemotherapy alone was effective in both RP and RPM models. Although tumors in both models responded well when exposed to cisplatin/etoposide, RPM tumors demonstrated a longer tumor response with a median survival of 83 days compared to 41 days in controls (*p* < 0.0001), while tumor-bearing RP mice had a median survival of 73.5 days, as compared to 53 days in controls (*p =* 0.0015). Strikingly, while ICI alone did not affect survival in RP mice, it significantly improved survival in RPM mice, with a median survival of 51.5 days compared to 41 days in controls (*p =* 0.0422). Similar to chemotherapy alone, the combination of cisplatin/etoposide with an anti-PD-1 antibody was effective in both mouse models, with RPM tumors showing higher sensitivity (Fig. 3b). Our findings suggest that MMR deficiency sensitizes SCLC to ICI therapy.

## Discussion

Our study assesses the role of *Msh2* deletion in driving tumorigenesis, genomic instability, and response to ICI in a novel mouse model of SCLC. By incorporating a conditional *Msh2* knockout allele into the RP model, we generated a preclinical model (RPM) that mimics the MMR mutational signature and high TMB as observed in 15% of SCLC clinical cases in a recent study (Liu et al. 2024). Unlike previous preclinical tools, our system enables the study of therapeutic response dynamics in the presence of substantial mutational rates driven by an MMR defect, which is also detected in a subset of human SCLC (Liu et al. 2024).

Our findings uncovered that RPM tumors exhibit accelerated aggressiveness compared to RP lesions. Despite a delayed onset of the tumor formation, RPM-depleted tumors led to earlier death. We speculate that *Msh2* deletion accelerates tumor progression, a finding that aligns with the role of MMR deficiency in promoting genomic instability (Dietlein et al. 2014a). This insight, combined with existing literature on the contribution of MMR deficiency to poor prognosis and aggressive tumor behavior in various cancers, such as colorectal malignancies (Oliveira et al. 2019), underscores the potential clinical relevance of our research.

WES analysis on micro-dissected lung tumors revealed a marked increase in TMB and mutational signatures associated with MMR in RPM tumors with homozygous *Msh2* deletion (*Msh2*^*fl/fl*^). The relationship between MMR deficiency and high TMB is well documented, with studies showing that MSI, a form of genetic hypermutability resulting from impaired *Msh2*, causes the accumulation of very high numbers of somatic mutations (Popat et al. 2005b). Moreover, the *Msh2* deletion in the RP background significantly amplified the load of predicted neoantigens, suggesting a stronger potential to elicit antitumor immune responses and heightened susceptibility to ICI.

Furthermore, as TMB and MSI-high have been proposed as potential predictive biomarkers for ICI response in SCLC (Taniguchi et al. 2020), we designed in vivo treatment regimens with a PD-1 blocking antibody alone or in combination with platinum-based chemotherapy, mirroring the clinical standard of care. We observed that while chemotherapy alone or in combination with ICI was beneficial in both mouse systems, only the RPM mice displayed increased overall survival when exposed to ICI alone. This notable response rate, which could be attributed to the elevated mutational load, aligns with recent clinical data indicating that high TMB bolsters the efficacy of immune checkpoint inhibition in SCLC patients (Hellmann et al. 2018). Previous studies have also reported a correlation between MMR-deficient/MSI-high mutational landscapes and enhanced ICI benefits in patients with colon cancer (Le et al. 2015; Olivares-Hernández et al. 2022). Notably, the role of MMR genes has been recently explored in non-small cell lung cancer (NSCLC), and alterations were associated with improved efficacy of nivolumab (a PD-1 checkpoint inhibitor) (Rizvi et al. 2015). However, evidence supporting the inclusion of TMB and MMR defects as ICI predictors in clinical practice is insufficient and contradictory. To illustrate, Westcott et al. (2023) reported that MMR deficiency developed in similar in vivo lung and colon cancer models failed to display increased ICI response due to pronounced intratumor heterogeneity of mutations. In addition, in our study, combining an anti-PD-1 antibody with chemotherapy in RPM tumors did not outperform chemotherapy alone (Fig. 3b). There are a number of possible explanations for this. First, the chemotherapy backbone may simply induce toxicities in the T cell compartment, which prevent efficient T cell-mediated tumor eradication. However, given that numerous chemotherapy/immune checkpoint inhibitor combinations were shown to increase efficacy, compared to chemotherapy alone (for instance cisplatin/etoposide/durvalumab in SCLC (Paz-Ares et al. 2019) or carboplatin/pemetrexed/pembrolizumab in NSCLC (Garassino et al. 2023), we believe that this explanation is unlikely. We rather expect that addition of immune therapy to the cisplatin/etoposide backbone increases overall toxicity, which may cover a potential survival benefit brought about by the addition of an immune checkpoint inhibitor. While we did not investigate any potential immune-mediated toxicities in our experimental animals, the occurrence of immune-related adverse events (irAEs) could restrict the effectiveness of the combined treatment (Postow et al. 2018).

While this study provides insights into the role of MMR deficiency on SCLC tumorigenesis and response to immunotherapy, there are several limitations to acknowledge. Despite effectively mirroring MMR deficiency observed in some human SCLC tumors, the RPM mouse model may not fully capture the genetic and phenotypic diversity of human SCLC. The artificial genetic manipulations in mouse models might not entirely reflect the spontaneous development of mutations in human cancers. In fact, our experimental mice are not exposed to cigarette smoke, which is the dominant carcinogen that drives human SCLC development. In addition, while *Msh2* deficiency clearly promotes and MMR deficiency phenotype, there are numerous additional genetic aberrations that drive an MMR deficiency phenotype (Reinhardt and Yaffe 2013; Dietlein et al. 2014b; Dietlein and Reinhardt 2014). Whether all of these genomic aberrations produce an identical genome maintenance defect is, however, unclear. Thus, our RPM approach likely only mimics a subset of MMR-defective SCLC cases. Furthermore, while our findings suggest enhanced sensitivity to immune checkpoint inhibitors in MMR-deficient SCLC, further studies are needed to validate our findings and assess potential toxicities of such a therapeutic approach and evaluate the long-term efficacy and potential resistance mechanisms in a clinical setting. It is worth noting that the experimental schemes used in this study, including the genetic engineering of mice and specific immunotherapy regimens, do not comply with standard clinical practice. This presents a challenge in translating our results to human patients. These limitations highlight the need to deepen our understanding of the role of MMR deficiency in SCLC by using diverse models and complementary methodologies.

All together, our research suggests that our novel high TMB SCLC preclinical model, RPM, is a valuable tool for investigating therapeutic response dynamics *in vivo*. Particularly, this model holds the potential to deepen our understanding of the complex interplay among the *Msh2* deletion, elevated mutational load, and response to ICI. These insights could pave the way for potential stratification strategies for patients who would benefit from such treatment while minimizing unnecessary toxic effects on the remaining patients. Additionally, the RPM model may be valuable in developing and testing new treatment strategies targeting tumors with MSI/TMB-high tumors. Future efforts to validate our observations and identify the underlying mechanisms could significantly contribute towards personalized treatment approaches in SCLC, potentially improving treatment outcomes and optimizing therapeutic strategies.

## Conclusion

Our study introduces a novel genetically engineered mouse model that recapitulates MMR deficiency in SCLC (RPM). This model uncovers that MMR deficiency leads to increased tumor aggressiveness, elevated TMB, and enhanced response to ICI therapy. These results suggest that MMR deficiency could be considered a stratifying criterion for ICI treatment of SCLC patients. Further research and clinical validation are necessary to fully understand the implications of MMR deficiency in SCLC and improve patient outcomes.

## Electronic supplementary material

Below is the link to the electronic supplementary material.

Supplementary Material 1

## Data Availability

The datasets generated and/or analyzed in the current study are available from the corresponding author upon reasonable request.

## Abbreviations

COSMIC: Catalogue Of Somatic Mutations In Cancer
FELASA: Federation of European Laboratory Animal Science Associations
GATK: Genome Analysis ToolKit
GEMM: Genetically Engineered Mouse Model
HLA-I: Human Leukocyte Antigen Class I
ICI: Immune Checkpoint Inhibitor
IEDB: Immune Epitope Database
irAEs: Immune-related adverse events
IVC: Individually Ventilated Cages
LANUV: North Rhine-Westphalia State Agency for Nature, Environment, and Consumer Protection
MMR: Mismatch Repair
MRI: Magnetic Resonance Imaging
MSI: Microsatellite instability
PBS: Phosphate-Buffered Saline
RP: *Rb1*^*fl/fl*^;*Trp53*^*fl/fl*^
RPM: *Rb1*^*fl/fl*^;*Trp53*^*fl/fl*^;*Msh2*^*fl/fl*^
SCLC: Small Cell Lung Cancer
SNV: Single Nucleotide Variant
TMB: Tumor Mutational Burden
WES: Whole Exome Sequencing
