# Supplementary material for: A novel mouse model recapitulating the MMR-defective SCLC subtype uncovers an actionable sensitivity to immune checkpoint blockade
Source: J Cancer Res Clin Oncol. 2024 Nov 14;150(11):496. doi: 10.1007/s00432-024-05942-9 (PMC11564195; doi:10.1007/s00432-024-05942-9)
Supplement: Supplementary file 1 — Supplementary Material 1 [file 432_2024_5942_MOESM1_ESM.pdf]

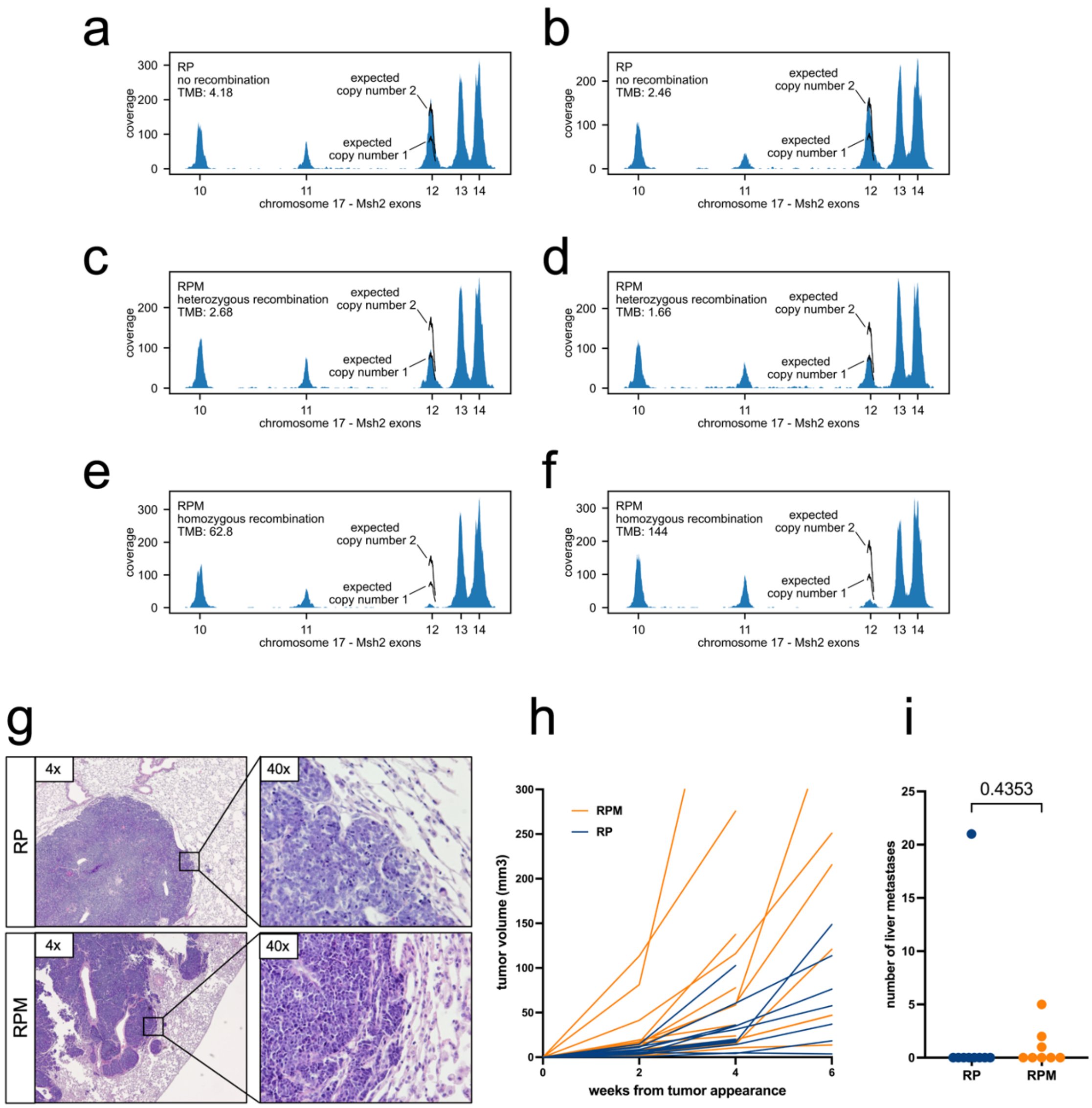

Extended Data Fig. 1 Characterization of RPM primary and secondary tumors

Exemplary coverage plots of tumors from RP and RPM genotypes. The black lines represent the predicted coverage profile of exon 12 in the absence of recombination (copy number = 2) or in the presence of heterozygous recombination (copy number = 1). **a, b** The coverage profile of *Msh2* exon 12 in two exemplary mice of the RP genotype fits the predicted coverage in the absence of recombination. **c, d** The coverage profile of *Msh2* exon 12 in two mice of the RPM genotype fits the predicted coverage in the presence of heterozygous recombination. **e, f** The coverage profile of *Msh2* exon 12 in two exemplary mice of the RPM genotype shows full recombination. Residual coverage within exon 12 is likely derived from low-level contamination from healthy lung cells. **g** Histological analysis of RPM lung tumors confirms typical human SCLC morphological features similar to RP. Exemplary hematoxylin and eosin staining images captured at 4x and 40x magnifications are shown. **h** Individual tumor growth trajectories over

the weeks from tumor detection determined by quantifying segmented tumors in MRI scans for n=12 RP and n=13 RPM lung lesions. **i** The number of liver metastases determined post-mortem in n=9 RP and n=8 RPM subjects. Mann-Whitney t-test (i).

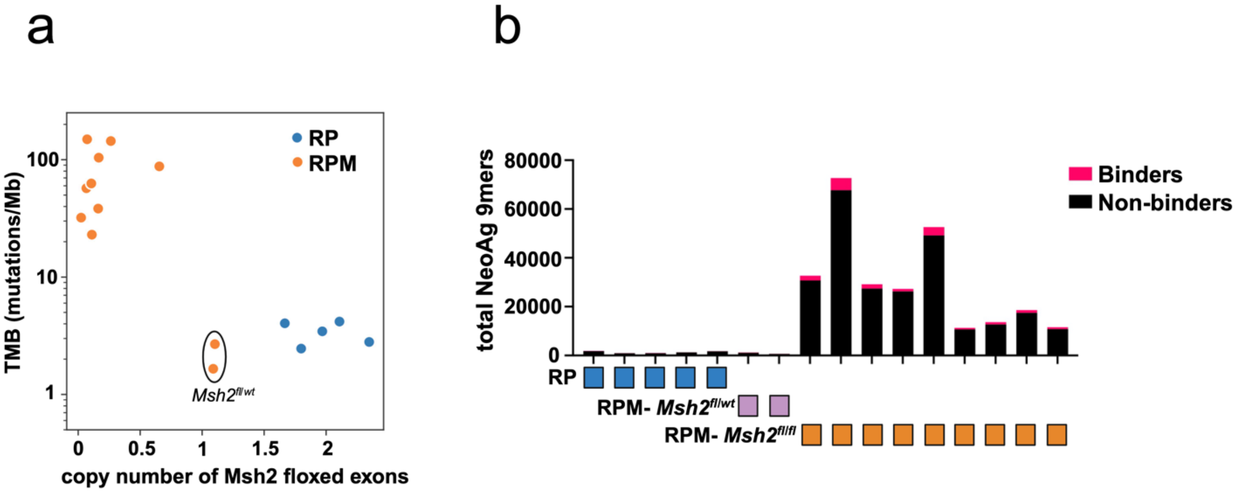

Extended Data Fig. 2 Genetic composition of RPM tumors based on whole exome sequencing (WES)

a Copy number analysis of *Msh2* floxed exons demonstrates homozygous and heterozygous loss in n=9 and n=2 RPM subjects, respectively, as well as no alteration in n=5 RP mice.
b Determination of binder and non-binder HLA-I peptide sequences of the most common HLA-I peptide pocket (9mers). All analyses are performed in n=5 RP, n=9 RPM with *Msh2*$^{fl/fl}$, and n=2 RPM with *Msh2*$^{fl/wt}$.

Extended Material and Methods

SCLC tumor induction

To initiate lung tumor formation, mice aged between eight and twelve weeks were exposed to an Adeno-Cre virus. The animals were first anesthetized through intraperitoneal injection of Ketavet (100 mg/kg) and Rompun (20 mg/kg). Subsequently, they received an intratracheal instillation of adenovirus expressing Cre-recombinase (Ad5-CMV-Cre, 2.5 x 107 PFU). Viral vectors were obtained by the University of Iowa Viral Vector Core (http://www.medicine.uiowa.edu/vectorcore).

MRI scans and tumor volume estimation

Five months after tumor induction, lung lesion development was assessed bi-weekly using magnetic resonance imaging (MRI). Imaging was conducted utilizing an Achieva 3.0T clinical system (Philips Healthcare, Best, the Netherlands) with a specialized mouse solenoid coil (Philips Healthcare, Hamburg, Germany). To acquire the scans, animals were anaesthetized using 2.5% isoflurane. T2-weighted MR images were generated in the axial plane using a turbospin echo (TSE) sequence (repetition time [TR] = 3819 ms, echo time [TE] = 60 ms, field of view [FOV] = 40 Å~ 40 Å~ 20 mm3, reconstructed voxel size = 0.13 Å~ 0.13 Å~ 1.0 mm3, number of average =1). The MR images, stored in DICOM files, were analyzed to identify and measure regions of interest (ROIs) using Horos software. For this, tumors were segmented in sequential scans, and the tumor volume was computed. The tumor volume progression was displayed as a fold change to correct for variance in tumor size at the start of the experiment. Statistical analyses were performed using the Mann-Whitney test.

Overall survival

To determine the role of the *Msh2* deletion in SCLC tumorigenesis, the overall survival of RPM mice was recorded and compared to RP mice. Upon MRI imaging confirmation, lesions with a 5-20 mm^3 volume at initial detection were considered for analysis. Tumor onset, survival probability from birth to mortality, and survival from tumor appearance to mortality were estimated. Statistical analyses were performed using the long-rank (Mantel-Cox) test.

Histology

To confirm typical SCLC histological features of RPM tumors, Hematoxylin/Eosin (H&E) staining was performed. Upon termination of RP and RPM animals, lung tumors were micro-dissected, fixed in a 4% formaldehyde solution (Merck), and embedded in paraffin. Five-micron tissue sections were collected using a microtome and subjected to H&E staining following standard protocols.
